# Operationalizing and Implementing Pretrained, Large Artificial Intelligence Linguistic Models in the US Health Care System: Outlook of Generative Pretrained Transformer 3 (GPT-3) as a Service Model

**DOI:** 10.2196/32875

**Published:** 2022-02-10

**Authors:** Emre Sezgin, Joseph Sirrianni, Simon L Linwood

**Affiliations:** 1 The Abigail Wexner Research Institute Nationwide Children's Hospital Columbus, OH United States; 2 School of Medicine University of California Riverside Riverside, CA United States

**Keywords:** natural language processing, artificial intelligence, generative pretrained transformer, clinical informatics, chatbot

## Abstract

Generative pretrained transformer models have been popular recently due to their enhanced capabilities and performance. In contrast to many existing artificial intelligence models, generative pretrained transformer models can perform with very limited training data. Generative pretrained transformer 3 (GPT-3) is one of the latest releases in this pipeline, demonstrating human-like logical and intellectual responses to prompts. Some examples include writing essays, answering complex questions, matching pronouns to their nouns, and conducting sentiment analyses. However, questions remain with regard to its implementation in health care, specifically in terms of operationalization and its use in clinical practice and research. In this viewpoint paper, we briefly introduce GPT-3 and its capabilities and outline considerations for its implementation and operationalization in clinical practice through a use case. The implementation considerations include (1) processing needs and information systems infrastructure, (2) operating costs, (3) model biases, and (4) evaluation metrics. In addition, we outline the following three major operational factors that drive the adoption of GPT-3 in the US health care system: (1) ensuring Health Insurance Portability and Accountability Act compliance, (2) building trust with health care providers, and (3) establishing broader access to the GPT-3 tools. This viewpoint can inform health care practitioners, developers, clinicians, and decision makers toward understanding the use of the powerful artificial intelligence tools integrated into hospital systems and health care.

## Introduction

In 2020, OpenAI unveiled their third-generation language generation model, which is known as the generative pretrained transformer 3 (GPT-3) model [1]. This model was the latest in a line of large pretrained models designed for understanding and producing natural language by using the transformer architecture, which was published only 3 years prior and significantly improved natural language understanding task performance over that of models built on prior architectures [2]. However, GPT-3’s development was remarkable because it resulted in a substantial increase in the model’s size; it increased by more than 10-fold in 1 year, reaching 175 billion weights [1-3]. GPT-3’s increased model size makes it substantially more powerful than prior models; propels its language capabilities to near–human-like levels; and, in some cases, makes it the superior option for several language understanding tasks [1].

Ordinarily, deep learning tasks require large amounts of labeled training data. This requirement usually limits the tasks to which deep learning can be effectively applied. However, with its increased model size, GPT-3 has an enhanced capability for so-called *few-shot*, *one-shot*, and *zero-shot* *learning* when compared to prior models [1,4]. These learning methods involve training a model on significantly smaller amounts of training data. In these methods, the models are given a description of the task and, if applicable, a handful of examples to learn from, with few-shot training on only hundreds to thousands of instances, one-shot training on only 1 example, and zero-shot training on only the task description.

GPT-3 was designed as a language generation model, focusing on producing appropriate text responses to an input. Although it can be adapted to address more traditional machine learning tasks, such as answering yes-no questions, matching pronouns to their nouns, and conducting sentiment analyses [1], GPT-3’s text generation capabilities have attracted much attention as a potential solution for a variety of problems, such as creating enhanced chatbots [5], answering complex questions, generating computer code from a design specification [6], and writing news articles [7]. As such, there is much research on and commercial interest in using GPT-3 for a variety of text generation applications. Textbox 1 illustrates the power of GPT-3 in 3 question and answer examples [8]. In 3 cases, GPT-3 provided human-like logical and intellectual responses. However, applying GPT-3 in health care is still an emerging topic.

A plethora of research articles have outlined the potential opportunities for integrating artificial intelligence (AI) and machine learning models in a variety of hospital and health care settings [9-11]. However, the authors also quickly pointed out the many development and implementation challenges to deploying such solutions, such as the lack of transparency [11], potential model biases [9], and financing [10]. A successful deployment of GPT-3 would need to overcome all of these standard barriers to AI implementation alongside the additional challenges that GPT-3 itself introduces [12]. In this viewpoint, we introduce the use of GPT-3 in the health care setting with a use case and discuss implementation and operational considerations for its use in clinical practice.

**Generative pretrained transformer 3 (GPT-3) question and answer examples.**

**Question and answer**

- Human-generated question: “What is human life expectancy in the United States?”
- GPT-3–generated response: “Human life expectancy in the United States is 78 years.”

**Text summary**

- Human-generated prompt: “Jupiter is the fifth planet from the Sun and the largest in the Solar System. It is a gas giant with a mass one-thousandth that of the Sun, but two-and-a-half times that of all the other planets in the Solar System combined. Jupiter is one of the brightest objects visible to the naked eye in the night sky, and has been known to ancient civilizations since before recorded history. It is named after the Roman god Jupiter. When viewed from Earth, Jupiter can be bright enough for its reflected light to cast visible shadows, and is on average the third-brightest natural object in the night sky after the Moon and Venus.”
- GPT-3–generated response (a second grader’s language): “Jupiter is a huge planet made of gas. It is the fifth planet from the sun. It is very big and bright. It is the third brightest object in the night sky after the moon and Venus. Jupiter has many moons. One of the moons is bigger than the planet Mercury.”

**A chatbot (sarcastic)**

- Human: “Why is the sky blue?”
- GPT-3 chatbot: “I’m not sure. I’ll ask my friend Google.”

## A Use Case: GPT-3 in Health Care

**Figure 1:**
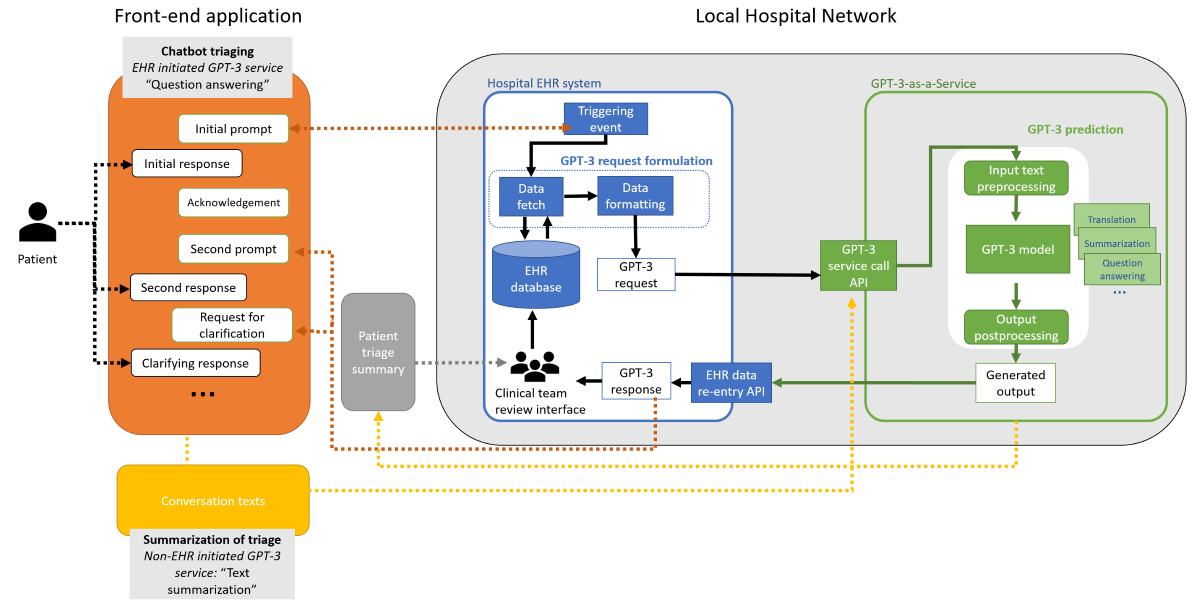
GPT-3 use case (chatbot triaging and patient note summarization). API: application programming interface; EHR: electronic health record; GPT-3: generative pretrained transformer 3.

The power of meaningful text generation by GPT-3 makes it an ideal tool for human-machine conversations, especially those conducted via chatbots. In our case, we illustrate the use of GPT-3 within a hospital network. In this hypothetical example, the hospital is providing a chatbot triaging mechanism for incoming patients to reduce the overhead at clinics and increase the safety and quality of care during the COVID-19 pandemic. The chatbot has to be connected to the hospital network, combined with a triage text summary service that is to be reviewed, and stored in the electronic health record (EHR; Figure 1). Putting aside the front-end details in this workflow (Figure 1), this use case outlines a typical implementation of GPT-3 as a service within a health system.

In this example, triage could be initiated by a patient or a hospital to conduct a health screening. The front-end application is operationalized through a chatbot mechanism over a front-end application, which could be a patient portal app, voice assistant, phone call, or SMS text messaging. Once a connection is established, the hospital system formulates GPT-3 requests by gathering patient health information and formatting this information to be interpretable with the GPT-3 model. Within the secure hospital network, GPT-3 is located outside of the EHR and provided as the “GPT-3-as-a-Service” platform. The application programming interface enables interoperability and acts as a gatekeeper for the data transfer of requests and responses. Once a request is received, the “GPT-3-as-a-Service” platform preprocesses the data and requests, allocates the tasks to be completed, produces outputs in an interpretable format, and sends the outputs to users. The type of tasks allocated depends on the requests, which, in our case, are question answering, text generation or culturally appropriate language translation, and text summarization. The response is sent back to the EHR system and then to the front-end application. At the end of triage, similar to the after-visit summary, the conversation text is summarized. To reduce the additional clinical burden of reading the whole conversation, GPT-3 summarizes the text (similar to a digital scriber) and stores it in the patient's health records. To avoid or address potential biases [12], correct errors, and increase the control over patient data use and the model, the human-in-the-loop model [13] can be implemented by using a report back mechanism at the front end, or the clinical team can be given oversight of GPT-3 integrated process in the hospital EHR system at the back end. Furthermore, the error corrections and adjustments in the text can be used to fine-tune the GPT-3 model to increase its accuracy and effectiveness.

To be able to execute this use case in a real-world setting, health care practitioners and decision makers should consider and address the following operational and implementation challenges.

## Implementation Considerations

### Processing Needs and Information Systems Infrastructure

Unlike more traditional AI models, GPT-3 is considerably larger in terms of memory requirements and is more computationally intensive. Specialized hardware for model training and execution—either graphics processing units or tensor processing units—is required for a scalable implementation. For any hospital system, additional investments for infrastructure to compensate for processing needs could be required.

Given its size, dependencies, and hardware requirements, a GPT-3 solution would likely need to be run as a service. For this service, hospital systems would need to submit a service request to the GPT-3 solution service, which would process the request and return its results back to the hospital system. The hospital local network in Figure 1 shows a sample workflow diagram for such an implementation. Such a setup would require diligent and significant provisioning, networking, and monitoring to ensure that the services are accessible and provide meaningful value.

### Operating Cost

Given the current state of hospital networks and EHR systems, the integration of GPT-3 solutions would require complex systems and high technical knowledge for effective deployment and be costly to operationalize. One possible solution to ease the burden of GPT-3 deployments is integration with cloud computing platforms within hospital systems. Many cloud computing providers offer the specialized hardware needed to run such models and can easily handle off-the-shelf networking and dynamic load balancing. This would ease the burden of the major components of GPT-3 deployment; however, outsourcing cloud computing platforms can potentially increase the operating cost.

### Model Bias

Several sources of bias can manifest themselves in a GPT-3–powered solution at different levels. At a model level, GPT-3 is trained on a large data set that has many problematic characteristics related to racial and sexist stereotypes, and as a result, the model learns certain biases against marginalized identities [14,15]. These biases, which are present in GPT-3, can be harmful in clinical settings. Korngiebel and Mooney [12] highlight the risks of using GPT-3 in health care delivery, noting specific examples where GPT-3 parrots extremist language from the internet [16] and affirms suicidal ideation [17].

Aside from the inherent bias of GPT-3’s initial training, fine-tuning on medical data could also introduce the unintentional biases present in historic medical data. Practical biases, such as the undertesting of marginalized subpopulations, can influence underlying clinical data and introduce bias during the training of predictive models [9]. Additionally, the implicit biases of health care professionals can influence diagnoses and treatments and are reflected in clinical notes [18], which, if used to fine-tune GPT-3, would potentially affect the developed model.

Given these biases, it would be unwise to deploy GPT-3 or any other sizable language model without active bias testing [15]. Explicit procedures should be put in place to monitor, report, and react to potential biases produced by GPT-3 predictions. These mechanisms would ensure that GPT-3 can be used effectively without introducing harm to the patient. In our use case (Figure 1), we also added a human-in-the-loop mechanism, which can mandate the control, assessment, and training protocols and yield interpretable and manageable results.

### Evaluation Metrics

Aside from physical implementation, there are methodological considerations for deploying GPT-3. As Watson et al [10] notes in their investigation of model deployment in academic medical centers, clinical utility is a major concern for institutions. Understanding the best way to receive and interpret model results is imperative for a successful deployment, and ideally, model performance should be tracked and assessed by using evaluation methodologies and frameworks.

The evaluation of text generation tasks, that is, those that GPT-3 is designed to address, is notoriously difficult. Standard metrics, such as prediction sensitivity and positive predictive value, do not cleanly reflect correctness in text generation, as ideas can be expressed in many ways in text. More specialized text generation metrics, such as BLEU (Bilingual Evaluation Understudy) [19] and METEOR (Metric for Evaluation of Translation with Explicit Ordering) [20], try to account for text variation but still only examine text at a word level without capturing the fundamental meaning. Methods that do try to incorporate the meaning of text in text evaluation rely on other black-box deep learning models to produce a value [21]. Relying on a black-box evaluation method to evaluate a black-box model does not increase interpretability. Such a method would only result in lower trust overall and thus decrease the likelihood of the model being deployed.

Health care–specific evaluation methods and frameworks for text generation tasks are therefore needed. The development of more robust methodologies for evaluating text generation tasks in the health care domain is required before the significant adoption of GPT-3 technology can be achieved. It is imperative that data scientists, informaticists, developers, clinicians, and health care practitioners collaborate in the development of evaluation measures to ensure a successful implementation of GPT-3.

## Operational Considerations: Compliance, Trust, and Access

**Figure 2:**
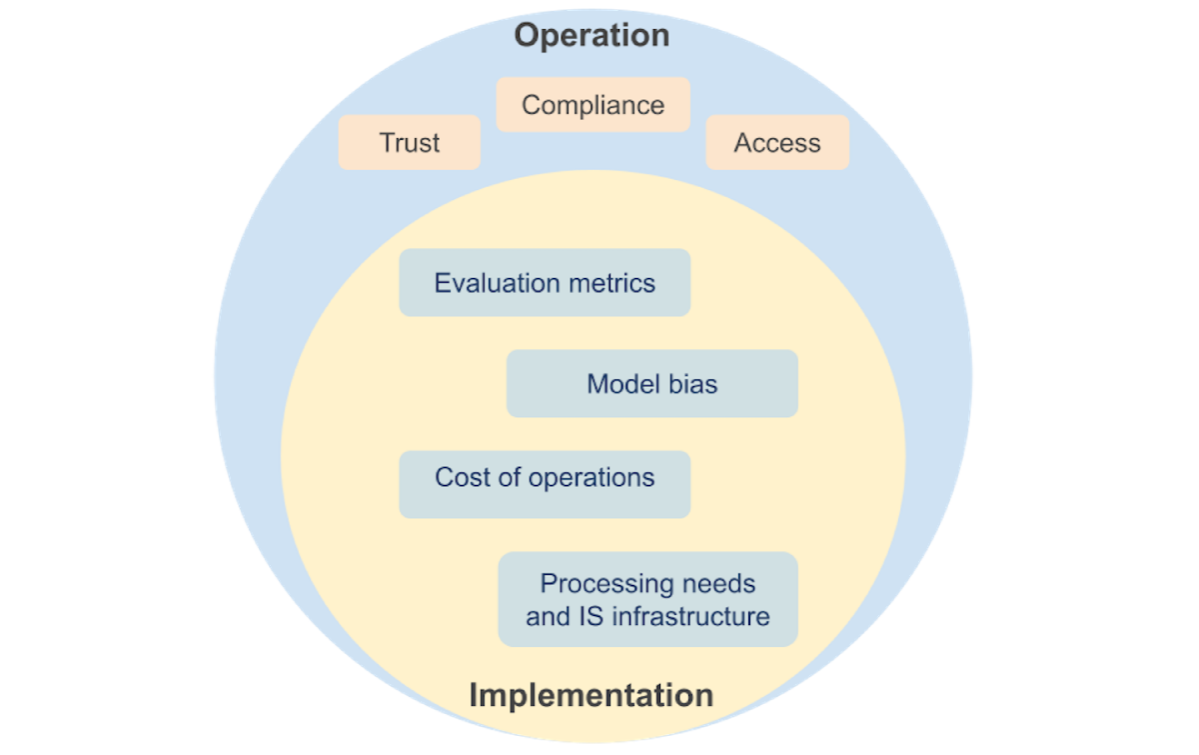
A model of operational and implementation considerations for generative pretrained transformer 3. IS: information systems.

In addition to implementation, there are 3 major operational factors driving the adoption of GPT-3 in health care, as follows: (1) GPT-3 needs to work in compliance with the Health Insurance Portability and Accountability Act (HIPAA), (2) technology providers need to earn trust from health care providers, and (3) technology providers should improve access to the tool (Figure 2).

Similar to GPT-3, there was huge enthusiasm to use the Amazon Alexa (Amazon.com Inc) voice assistant in health care delivery when it was released in 2014. However, at the time, Alexa was not yet legally able to store or transmit private health information. It took Amazon 5 years to become HIPAA compliant and to be able to sign business associate agreements with health care providers [22]. A limited number of Alexa skills was released, and there is still a long list of other Alexa skills waiting to become HIPAA compliant. This example shows the slow progress of legislation changes and regulation updates for including new technologies in health care, suggesting that efforts should be put forward as early as possible for GPT-3. Without HIPAA compliance, the adoption of GPT-3 in health care can be a false start [23]. However, although HIPAA compliance may not be immediate, it may be gradually progressing. GPT-3 is a black-box model, which complicates the HIPAA compliance process because unlike with other types of programmatic solutions, it is harder to decipher how data are processed internally by the model itself. However, assuming that GPT-3 will be deployable in the future, operations will start with implementing the limited capabilities of GPT-3 (ie, storing and transmitting data, running behind the firewalls of specific hardware [security rules], and analyzing a specific data set or patient cohort [privacy rules]). In parallel, further practices are needed to optimize the payment models for accommodating GPT-3 and seek opportunities for satisfying the US Food and Drug Administration’s requirements for software as a medical device [24] with regard to using AI in clinical applications.

In addition to legal requirements, trust must be established among patients, health care providers, and technology companies to adopt GPT-3 [25]. It is common for technology companies to claim the right that they can use their customers’ data to further improve their services or achieve additional commercial value. Additionally, the culture of skepticism toward AI among clinicians can place a heavy burden on model interpretability and result in lower trust in clinical care than in other industries [10]. Unlike commercial implementations, GPT-3 needs to be explicitly discussed in terms of what it will and will not do with a patient’s data. Health care providers’ data governance committees need to be aware and comfortable when they sign the service agreement with GPT-3. Given the black-box nature of GPT-3, an operational strategic approach will be necessary for interpreting the evaluation reports and outcomes that are generated through the human-in-the-loop model.

Access also needs to be ensured. Training large language models like GPT-3 can cost tens of millions of dollars. As such, GPT-3 is innovating the business model of access. Currently, GPT-3 is privately controlled by OpenAI, and health care providers can remotely run the program and pay for usage per token (1000 tokens are approximately equivalent to 750 words) [26]. In September 2020, Microsoft bought an exclusive license to GPT-3, with plans to integrate it into its existing products. Similarly, a number of companies are already integrating GPT-3 model predictions into their products. However, this business model also limits open-access research and development and will eventually limit improvements, such as advancements in translation mechanisms and all-inclusive, equity-driven approaches in conversational agent development. In these early stages, open-source alternatives, such as GPT-J [27], may help health care developers and institutions assess operational viability. In future iterations, once the value of using GPT-3 in the health care setting is assured, the responsibility of accessibility could be delegated to health care and government agencies. Such agencies may distribute the “GPT-3-as-a-Service” platform through secure cloud platforms and establish a federated learning mechanism to run decentralized training services while collaboratively contributing to the GPT-3 model [28]. This would also reduce the burden on individual health systems when it comes to building, training, and deploying their own GPT-3 platforms and reduce costs. These advantages are especially beneficial for hospitals in low-resource settings.

## Conclusion

In this viewpoint, we briefly introduce GPT-3 and its capabilities and outline considerations for its implementation and operationalization in clinical practice through a use case. Building on top of Korngiebel and Mooney’s [12] remarks toward unrealistic, realistic, feasible, and realistic but challenging use cases, we provide consideration points for implementing and operationalizing GPT-3 in clinical practice. We believe that our work can inform health care practitioners, developers, clinicians, and decision makers toward understanding the use of the powerful AI tools integrated into hospital systems and health care.

## Abbreviations

AI: artificial intelligence
BLEU: Bilingual Evaluation Understudy
EHR: electronic health record
GPT-3: generative pretrained transformer 3
HIPAA: Health Insurance Portability and Accountability Act
METEOR: Metric for Evaluation of Translation With Explicit Ordering
PCORI: Patient-Centered Outcomes Research Institute
